# Supplementary material for: Generation of SARS-CoV-2 escape mutations by monoclonal antibody therapy
Source: Nat Commun. 2023 Jun 7;14:3334. doi: 10.1038/s41467-023-37826-w (PMC10246534; doi:10.1038/s41467-023-37826-w)
Supplement: Supplementary file 1 — Supplementary information [file 41467_2023_37826_MOESM1_ESM.pdf]

**Supplementary Materials**

# Generation of SARS-CoV-2 escape mutations by monoclonal antibody therapy

Manon Ragonnet-Cronin[1,2*#], Rungtiwa Nutalai[3*], Jiandong Huo[4*#], Aiste Dijokaite-Guraliuc[3*], Raksha Das[3], Aekkachai Tuekprakhon[3], Piyada Supasa[3], Chang Liu[3,5], Muneeswaran Selvaraj[3], Natalie Groves[1], Hassan Hartman[1], Nicholas Ellaby[1], J. Mark Sutton[1], Mohammad W. Bahar[4], Daming Zhou[4,5], Elizabeth Fry[4], Jingshan Ren[4], Colin Brown[1], Paul Klenerman[6,7,8,9], Susanna J. Dunachie[6,7,9], Juthathip Mongkolsapaya[3,10],Susan Hopkins[1], Meera Chand[1], David I. Stuart[4#^], Gavin R. Screaton[3#*] and Sakib Rokadiya[1#*]

1. Genomics Public Health Analysis, UK Health Security Agency

2. Centre for Global Infectious Disease Analysis, Imperial College London

3. Wellcome Centre for Human Genetics, Nuffield Department of Medicine, University of Oxford, Oxford, UK

4. Division of Structural Biology, Nuffield Department of Medicine, University of Oxford, The Wellcome Centre for Human Genetics, Oxford, UK

5. Chinese Academy of Medical Science (CAMS) Oxford Institute (COI), University of Oxford, Oxford, UK

6. Nuffield Department of Medicine, University of Oxford, Oxford, UK

7. Oxford University Hospitals NHS Foundation Trust, Oxford, UK

8. Translational Gastroenterology Unit, University of Oxford, UK

9. NIHR Oxford Biomedical Research Centre, University of Oxford, Oxford, UK

10. Mahidol-Oxford Tropical Medicine Research Unit, Bangkok, Thailand, Department of Medicine, University of Oxford, Oxford, UK

**Figure S1. P-values for differences in spike (S) amino acid frequencies between pre- and post-treatment sequences.** The indicated cut off dates, following the mAb treatment were used for the acquisition of the post treatment sample. p values were calculated using a one-sided Fisher's test. No adjustments were made for multiple comparisons.  All exact p values are provided in the source data for this figure.

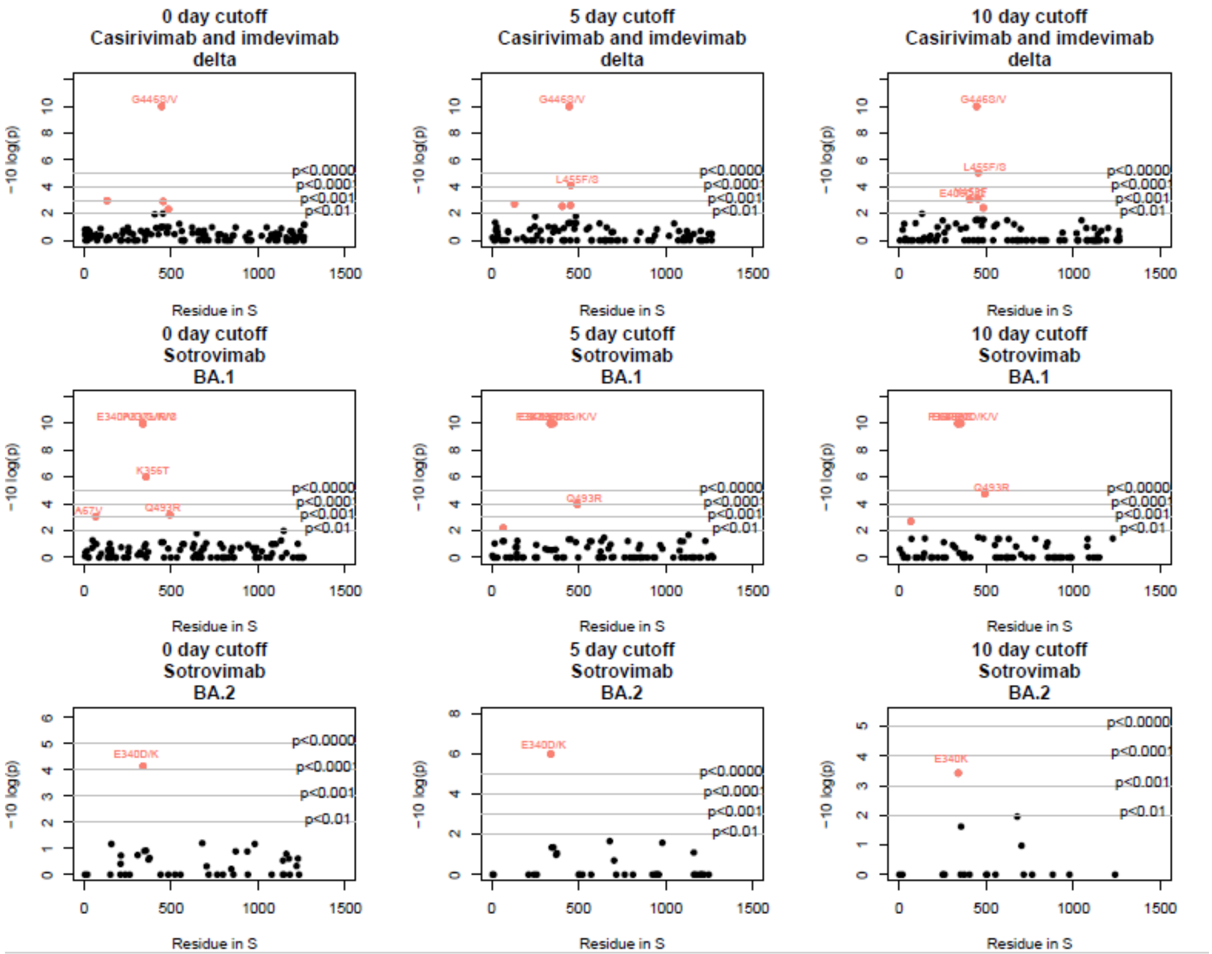


**Figure S2. Surface plasmon resonance (SPR) analysis of interaction between Delta and BA.1 RBD mutants and therapeutic mAbs.** (A-D; F-G; I, L, N, P) Sensorgrams showing the binding of wild-type Delta RBD and Delta RBD mutants to casirivimab/imdevimab, with affinity and kinetic parameters shown. (E, J, K, M, O) 1:1 binding equilibrium analysis of binding of Delta RBD mutants to casirivimab/imdevimab, with affinity values shown. (H) Binding of Delta RBD+G446V to imdevimab is severely reduced compared to that of wild-type Delta RBD, so that the binding could not be

accurately determined, as shown by a single-injection of 1 μM RBD over sample flow cells containing imdevimab. Related to **Figure S3** and **Table 3**.

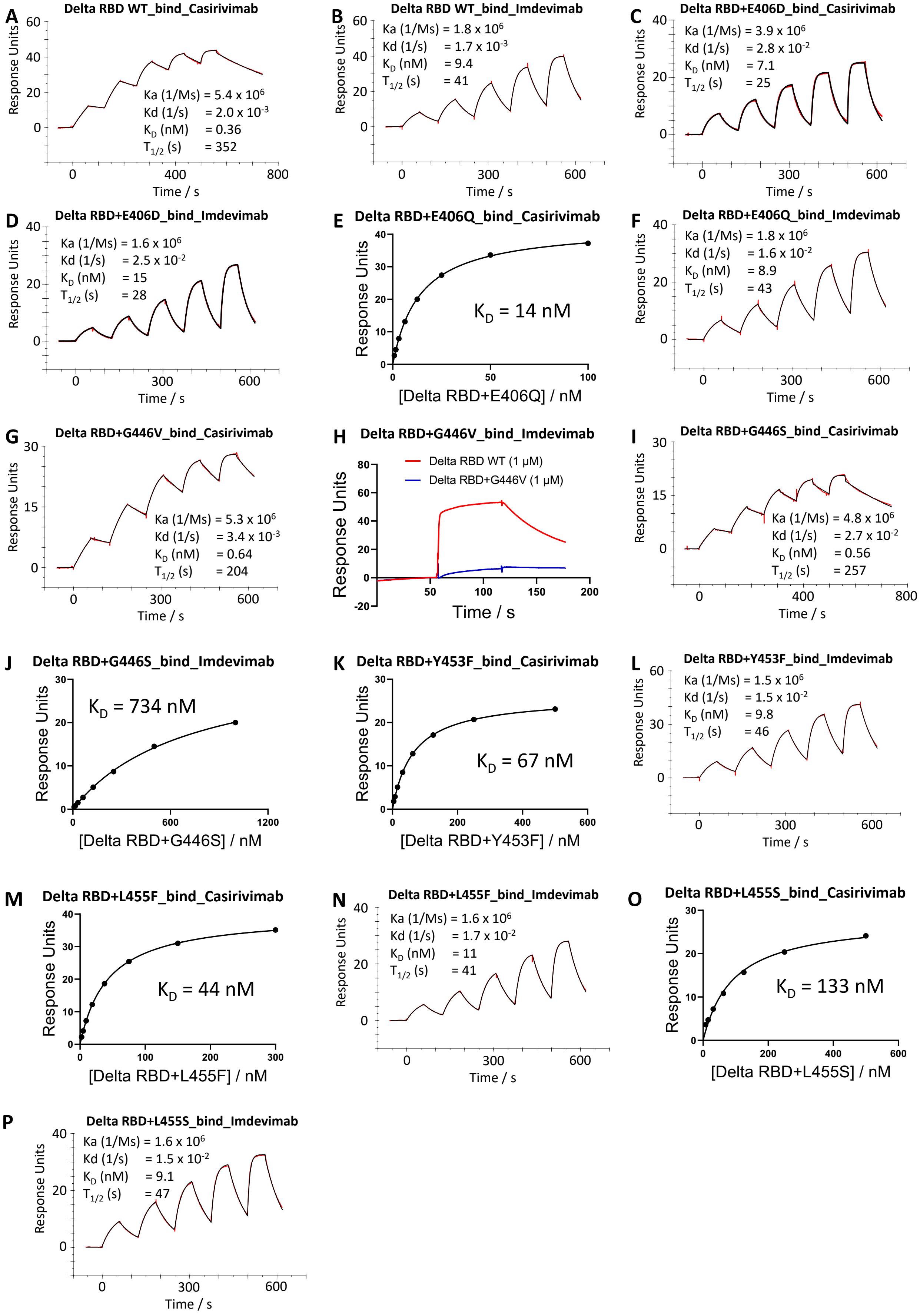

**Figure S3. Surface plasmon resonance (SPR) analysis of interaction between Delta and BA.1 RBD mutants and therapeutic mAbs.** (A, C) 1:1 binding equilibrium analysis of binding of Delta RBD mutants to casirivimab, with affinity values shown. (B, D) Binding of Delta RBD+G446V+Y453F and Delta RBD+G446V+L455F to imdevimab is severely reduced compared to that of wild-type Delta RBD, so that the binding could not be accurately determined, as shown by a single-injection of 1 μM RBD over sample flow cells containing imdevimab. (E) Sensorgram showing the binding of wild-type BA.1 RBD to sotrovimab, with affinity and kinetic parameters shown (published in Dejnirattisai et al., 2022). (F-K) 1:1 binding equilibrium analysis of binding of BA.1 RBD mutants to sotrovimab, with affinity values shown. Related to **Figure S2** and **Table 3**.

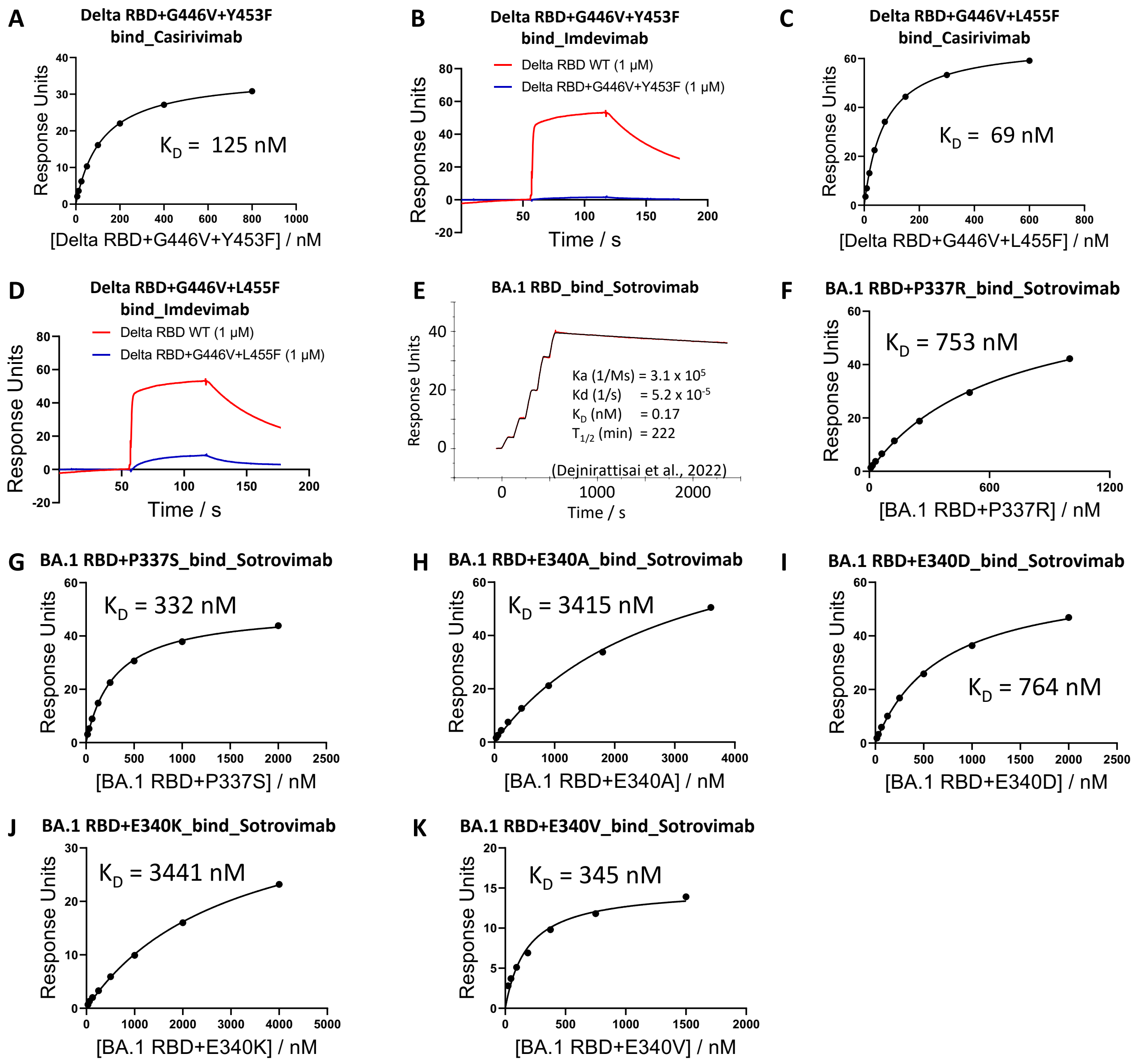

**Table S1. Dataset sizes (number of sequences)**. Note that some patients received multiple courses of treatment and thus may be counted more than once in the table.

| Treatment | Variant | Number of sequences (pre-treatment) | Number of sequences (≥ 1 day post-treatment) | Number of sequences (≥ 5 day post-treatment) | Number of sequences (≥ 10 day post-treatment) |
|---|---|---|---|---|---|
| Casirivimab and imdevimab | BA.1 | 181 | 116 | 94 | 80 |
| Casirivimab and imdevimab | BA.2 | 0 | 14 | 14 | 14 |
| Casirivimab and imdevimab | delta | 2173 | 298 | 138 | 82 |
| Molnupiravir | BA.1 | 1642 | 181 | 109 | 70 |
| Molnupiravir | BA.2 | 285 | 17 | 11 | 8 |
| Molnupiravir | delta | 29 | 8 | 7 | 3 |
| Paxlovid | BA.1 | 329 | 19 | 8 | 4 |
| Paxlovid | BA.2 | 698 | 41 | 15 | 10 |
| Paxlovid | delta | 0 | 2 | 2 | 2 |
| Remdesivir | BA.1 | 1268 | 529 | 371 | 307 |
| Remdesivir | BA.2 | 249 | 106 | 83 | 75 |
| Remdesivir | delta | 4779 | 955 | 402 | 224 |
| Sotrovimab | BA.1 | 3959 | 531 | 318 | 211 |
| Sotrovimab | BA.2 | 1656 | 130 | 49 | 25 |
| Sotrovimab | delta | 36 | 8 | 6 | 3 |

**Table S2. Treatment/gene/variant combinations tested**

| **Treatment** | **Gene** | **Variants** |
|---|---|---|
| Sotrovimab | *spike* | Delta, BA.1, BA.2 |
| Casirivimab and imdevimab | *spike* | Delta, BA.1, BA.2 |
| Remdesivir | *nsp7, nsp8, nsp9, nsp10, nsp12* | Delta, BA.1, BA.2 |
| Molnupiravir | *nsp7, nsp8, nsp9, nsp10, nsp12* | Delta, BA.1, BA.2 |
| Paxlovid | *nsp5* | Delta, BA.1, BA.2 |

## Table S3. Template, primers and expression vectors used for cloning of each RBD.

| RBD construct | Template (pseudovirus plasmid) | PCR primer sequence (5'to 3') | vector |
|---|---|---|---|
| Delta+E406D | Delta+E406D | F: CAGCTCCTGGGCAACGTGCT<br>R: CGTAAAAGGAGCAACATAG | pNEO |
| Delta+E406Q | Delta+E406Q | | |
| Delta+G446S | Delta+G446S | | |
| Delta+G446V | Delta+G446V | | |
| Delta+Y453F | Delta+Y453F | | |
| Delta+L455F | Delta+L455F | | |
| Delta+L455S | Delta+L455S | | |
| Delta+G446V+Y453F | Delta+G446V+Y453F | | |
| BA.1+P337R | BA.1+P337R | | |
| BA.1+P337S | BA.1+P337S | | |
| BA.1+E340A | BA.1+E340A | | |
| BA.1+E340D | BA.1+E340D | | |
| BA.1+E340K | BA.1+E340K | | |
| BA.1+E340V | BA.1+E340V | | |
| BA.1+K356T | BA.1+K356T | | |
| BA.1+R493Q | BA.1+R493Q | | |

## Table S4. Primers for pseudoviruses

| Primer | Sequence (5'to 3') |
|---|---|
| **Delta variants** | |
| **Delta+E406D_F** | GACAGCTTCGTGATCAGAGGCGACGACGTGAGACAGATCGCGCCAGGG |
| **Delta+E406D_R** | CCCTGGCGCGATCTGTCTCACGTCGTCGCCTCTGATCACGAAGCTGTC |
| **Delta+E406Q_F** | GACAGCTTCGTGATCAGAGGCGACCAAGTGAGACAGATCGCGCCAGGG |
| **Delta+E406Q_R** | CCCTGGCGCGATCTGTCTCACTTGGTCGCCTCTGATCACGAAGCTGTC |
| **Delta+G446S_F** | GAACTCTAACAATCTAGATTCGAAAGTTAGCGGCAATTACAATTACCTGTAC |
| **Delta+G446S_R** | GTACAGGTAATTGTAATTGCCGCTAACTTTCGAATCTAGATTGTTAGAGTTC |
| **Delta+G446V_F** | TAACAATCTAGATTCGAAAGTTGTAGGCAATTACAATTACCTGTAC |
| **Delta+G446V_R** | GTACAGGTAATTGTAATTGCCTACAACTTTCGAATCTAGATTGTTA |
| **Delta+Y453F_F** | GGCAATTACAATTACCGGTTCAGACTGTTCAGAAAGAGC |
| **Delta+Y453F_R** | GCTCTTTCTGAACAGTCTGAACCGGTAATTGTAATTGCC |
| **Delta+L455F_F** | GGCAATTACAATTACCGGTACAGATTCTTCAGAAAGAGCAATCTGAAGCC |
| **Delta+L455F_R** | GGCTTCAGATTGCTCTTTCTGAAGAATCTGTACCGGTAATTGTAATTGCC |
| **Delta+L455S_F** | GGCAATTACAATTACCGGTACAGAAGCTTCAGAAAGAGCAATCTGAAGCC |
| **Delta+L455S_R** | GGCTTCAGATTGCTCTTTCTGAAGCTTCTGTACCGGTAATTGTAATTGCC |
| **Delta+G446V+Y453F_F** | GTAGGCAATTACAATTACCGGTTCAGACTGTTCAGAAAGAGC |
| **Delta+G446V+Y453F_R** | GAACCGGTAATTGTAATTGCCTACAACTTTCGAATCTAGATTG |
| **BA.1 variants** | |
| **BA.1+P337R_F** | ATCACCAATCTGTGCCGTTTCGACGAGGTGTTC |
| **BA.1+P337R_R** | CCTCGTCGAAACGGCACAGATTGGTGATATTAG |
| **BA.1+P337S_F** | TCACCAATCTGTGCAGTTTCGACGAGGTGTTCAATG |
| **BA.1+P337S_R** | CACCTCGTCGAAACTGCACAGATTGGTGATATTAG |
| **BA.1+E340A_F** | CTGTGCCCTTTCGACGCGGTGTTCAATGCCAC |
| **BA.1+E340A_R** | GTGGCATTGAACACCGCGTCGAAAGGGCACAG |
| **BA.1+E340D_F** | GTGCCCTTTCGACGATGTGTTCAATGCCACC |
| **BA.1+E340D_R** | GGTGGCATTGAACACATCGTCGAAAGGGCAC |
| **BA.1+E340K_F** | CAATCTGTGCCCTTTCGACAAGGTGTTCAATGCCAC |
| **BA.1+E340K_R** | GTGGCATTGAACACCTTGTCGAAAGGGCACAGATTG |
| **BA.1+E340V_F** | GCGTAGCTGAAACCGGCACCAATCTGTGCCCTTTCGACGTGGTGTTCAATGCCACCAG |
| **BA.1+E340V_R** | CTGGTGGCATTGAACACCACGTCGAAAGGGCACAGATTGGTGCCGGTTTCAGCTACGC |
| **BA.1+K356T_F** | GCCAGCGTGTACGCATGGAACCGCACCCGGATAAGCAATTGCGTGGCC |
| **BA.1+K356T_R** | GGCCACGCAATTGCTTATCCGGGTGCGGTTCCATGCGTACACGCTGGC |
| **BA.1+R493Q_F** | GGCTTCAATTGCTACTTCCCTCTGCAGAGCTACTCGTTCAGACCTACC |
| **BA.1+R493Q_R** | GGTAGGTCTGAACGAGTAGCTCTGCAGAGGGAAGTAGCAATTGAAGCC |
| **pcDNA3.1 vector** | |
| **pcDNA3.1_BamHI_F** | GGATCCATGTTCCTGCTGACCACCAAGAG |
| **pcDNA3.1_Tag_S_EcoRI_R** | GAATTCTCACTTCTCGAACTGAGGGTGGC |
| **pcDNA3.1_Tag_S_EcoRI_F** | GCCACCCTCAGTTCGAGAAGTGAGAATTC |
| **pcDNA3.1_BamHI_R** | CTCTTGGTGGTCAGCAGGAACATGGATCC |
