# Supplementary material for: Generation of SARS-CoV-2 escape mutations by monoclonal antibody therapy
Source: Nat Commun. 2023 Jun 7;14:3334. doi: 10.1038/s41467-023-37826-w (PMC10246534; doi:10.1038/s41467-023-37826-w)
Supplement: Supplementary file 3 — Description of Additional Supplementary Files [file 41467_2023_37826_MOESM3_ESM.docx]

Description of additional supplementary files

## Title: Supplementary Data 1: Genbank IDs (provided as a csv)

## Description: All the genbank IDs of sequences used in the study (with weblinks)
