# Supplementary material for: Generation of SARS-CoV-2 escape mutations by monoclonal antibody therapy
Source: Nat Commun. 2023 Jun 7;14:3334. doi: 10.1038/s41467-023-37826-w (PMC10246534; doi:10.1038/s41467-023-37826-w)
Supplement: Supplementary file 4 — Reporting summary [file 41467_2023_37826_MOESM4_ESM.pdf]

nature portfolio

Corresponding author(s): David Stuart

Last updated by author(s): Jan 26, 2023

Reporting Summary

Nature Portfolio wishes to improve the reproducibility of the work that we publish. This form provides structure for consistency and transparency in reporting. For further information on Nature Portfolio policies, see our Editorial Policies and the Editorial Policy Checklist.

Statistics

For all statistical analyses, confirm that the following items are present in the figure legend, table legend, main text, or Methods section.

n/a	Confirmed	
☐	☒	The exact sample size (*n*) for each experimental group/condition, given as a discrete number and unit of measurement
☐	☒	A statement on whether measurements were taken from distinct samples or whether the same sample was measured repeatedly
☐	☒	The statistical test(s) used AND whether they are one- or two-sided<br>*Only common tests should be described solely by name; describe more complex techniques in the Methods section.*
☒	☐	A description of all covariates tested
☒	☐	A description of any assumptions or corrections, such as tests of normality and adjustment for multiple comparisons
☐	☒	A full description of the statistical parameters including central tendency (e.g. means) or other basic estimates (e.g. regression coefficient) AND variation (e.g. standard deviation) or associated estimates of uncertainty (e.g. confidence intervals)
☐	☒	For null hypothesis testing, the test statistic (e.g. *F*, *t*, *r*) with confidence intervals, effect sizes, degrees of freedom and *P* value noted<br>*Give P values as exact values whenever suitable.*
☒	☐	For Bayesian analysis, information on the choice of priors and Markov chain Monte Carlo settings
☒	☐	For hierarchical and complex designs, identification of the appropriate level for tests and full reporting of outcomes
☒	☐	Estimates of effect sizes (e.g. Cohen's *d*, Pearson's *r*), indicating how they were calculated

Our web collection on statistics for biologists contains articles on many of the points above.

Software and code

Policy information about availability of computer code

Data collection	Information on patients accessing covid treatment in the UK is collected by UKHSA. All patients on treatment are sequenced before they initiate treatment and regularly thereafter until they clear infection. Linkage to their sequence IDs is done within UKHSA. All sequences from patients known to have undergone treatment were downloaded from CLIMB. (https://www.climb.ac.uk/).
Data analysis	Analysis was performed though a series of scripts available on github (https://github.com/manonr/covid-therapeutics). Downloaded sequences were already aligned. Genome alignments were split into gene regions (spike, NSP5, NSP7, NSP8, NSP9, NSP10, NSP12 and NSP14) and translated to amino acids for analysis (https://github.com/manonr/covid-therapeutics/blob/main/python/get_sarscov2_proteins.py). Patient sequences were linked to lineage (https://github.com/manonr/covid-therapeutics/blob/main/R/script1_prepData_wBA45.R). For each cutoff (t=1, 5, 10, 14), a statistical analysis was conducted to compare patient sequences collected before treatment and after the post-treatment cutoff (script2_aafreq_perDrug_perVar_v4_refConsensus.R). For gene/treatment combinations of interest, results were plotted for the manuscript (script3d_therapeutics_plots_specificCombos_ms.R). We generated counts of patients who acquired the treatment associated mutations (script6_mutation_treatment_counts_v2.R).

For manuscripts utilizing custom algorithms or software that are central to the research but not yet described in published literature, software must be made available to editors and reviewers. We strongly encourage code deposition in a community repository (e.g. GitHub). See the Nature Portfolio guidelines for submitting code & software for further information.

Data

Policy information about availability of data

All manuscripts must include a data availability statement. This statement should provide the following information, where applicable:

- Accession codes, unique identifiers, or web links for publicly available datasets
- A description of any restrictions on data availability
- For clinical datasets or third party data, please ensure that the statement adheres to our policy

A subset of the UK SARS-CoV-2 genomes are submitted to GISAID (gisaid.org). Information on clinical treatments at the individual level, linked to sequence ID, cannot be shared.

Human research participants

Policy information about studies involving human research participants and Sex and Gender in Research.

Reporting on sex and gender	Analysis was not disaggregated by sex.
Population characteristics	The only population characteristic examined within the manuscript is treatment. Patients included were all treated for their COVID infection with one of casirivimab+imdevimab, molnupiravir, pnirmatrelvir plus ritonavir (Paxlovid), remdesivir or sotrovimab.
Recruitment	The analysis includes all patients who had received treatment in the UK, for whom at least one sample had been collected by 12 April 2022 and for whom a viral genetic sequence was available.
Ethics oversight	UKHSA England Research Ethics and Governance Group

Note that full information on the approval of the study protocol must also be provided in the manuscript.

Field-specific reporting

Please select the one below that is the best fit for your research. If you are not sure, read the appropriate sections before making your selection.

☒ Life sciences ☐ Behavioural & social sciences ☐ Ecological, evolutionary & environmental sciences

For a reference copy of the document with all sections, see nature.com/documents/nr-reporting-summary-flat.pdf

Life sciences study design

All studies must disclose on these points even when the disclosure is negative.

Sample size	Our analysis was conducted weekly from early March, with the sample size growing each time as new patients initiated treatment and had their virus genetically sequenced. Potential treatment associated mutations were further evaluated in the laboratory. By April, nine amino acid residues displayed a significant ($p<0.001$) frequency change in post-treatment sequences compared to pre-treatment sequences, suggesting possible evidence of selection, and we considered the results to be worthy of publication.
Data exclusions	No data were excluded from the analysis
Replication	The analysis was observational and could not be replicated.
Randomization	Allocation was not random, the population was subdivided by patient group. we also included date of sample and date of treatment in our analysis. We would not expect treatment-associated mutations to be associated with any patient characteristic beyond their treatment, and therefore we did not include additional patient covariates in the analysis.
Blinding	Processing of data was conducted programmatically, with pre-treatment and post-treatment, and each treatment category, compared statistically. The researcher was not blinded but did make have any input into the process.

Reporting for specific materials, systems and methods

We require information from authors about some types of materials, experimental systems and methods used in many studies. Here, indicate whether each material, system or method listed is relevant to your study. If you are not sure if a list item applies to your research, read the appropriate section before selecting a response.

Materials & experimental systems

n/a	Involved in the study	
☐	☒	Antibodies
☐	☒	Eukaryotic cell lines
☒	☐	Palaeontology and archaeology
☒	☐	Animals and other organisms
☐	☒	Clinical data
☒	☐	Dual use research of concern

Methods

n/a	Involved in the study	
☒	☐	ChIP-seq
☒	☐	Flow cytometry
☒	☐	MRI-based neuroimaging

Antibodies

Antibodies used	Sotrovimab/S309: Vir mAbs, Cat#S309; Casirivimab & Imdevimab: Regeneron mAbs, Cat#REGN10933, and REGN10987
Validation	Therapeutic antibodies validated for clinical use.

Eukaryotic cell lines

Policy information about cell lines and Sex and Gender in Research

Cell line source(s)	HEK293T/17 cells, source ATCC, Cat#CRL-11268™, Expi293F™, source Thermo Fisher, Cat# A14527
Authentication	not authenticated
Mycoplasma contamination	Expi293F negative for mycoplasma, HEK293T not tested
Commonly misidentified lines (See ICLAC register)	No commonly misidentified cell lines were used in this study

Clinical data

Policy information about clinical studies

All manuscripts should comply with the ICMJEguidelines for publication of clinical research and a completedCONSORT checklist must be included with all submissions.

Clinical trial registration	*Provide the trial registration number from ClinicalTrials.gov or an equivalent agency.*
Study protocol	*Note where the full trial protocol can be accessed OR if not available, explain why.*
Data collection	*Describe the settings and locales of data collection, noting the time periods of recruitment and data collection.*
Outcomes	*Describe how you pre-defined primary and secondary outcome measures and how you assessed these measures.*
